# (*E*)-3-(2-Bromo­phen­yl)-1-(3,4-dimeth­oxy­phen­yl)prop-2-en-1-one

**DOI:** 10.1107/S1600536812006046

**Published:** 2012-02-17

**Authors:** Zhe Li, Yanan Wang, Kesong Peng, Lingzi Chen, Shenghui Chu

**Affiliations:** a Life Science College, Wenzhou Medical College, Wenzhou, Zhejiang Province 325035, People’s Republic of China; b School of Pharmacy, Wenzhou Medical College, Wenzhou, Zhejiang Province 325035, People’s Republic of China

## Abstract

The crystal structure of the title compound, C_17_H_15_BrO_3_, a chalcone derivative, exhibits two crystallographically independent mol­ecules per asymmetric unit showing an *E* conformation about the ethyl­ene double bond. In each mol­ecule, the two phenyl rings are almost coplanar: the mean planes make dihedral angles of 9.3 (2) and 19.4 (2)°. In the crystal, mol­ecules are linked through weak inter­molecular C—H⋯O hydrogen bonds.

## Related literature

For related structures, see: Wu *et al.* (2009 ▶, 2010 ▶, 2011*a* ▶,*b* ▶); Huang *et al.* (2010 ▶); Peng *et al.* (2010 ▶). For background to and applications of chalcones, see: Nielsen *et al.* (2005 ▶); Wu *et al.* (2010 ▶, 2011*a* ▶,*b* ▶).
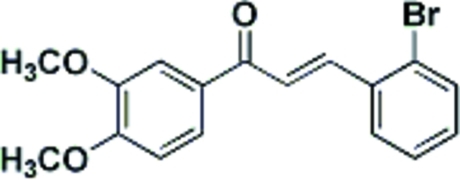


## Experimental

### 

#### Crystal data

C_17_H_15_BrO_3_
*M* *_r_* = 347.20
Triclinic, 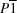

*a* = 11.574 (6) Å
*b* = 11.781 (6) Å
*c* = 11.877 (6) Å
α = 91.857 (9)°
β = 107.021 (9)°
γ = 91.917 (9)°
*V* = 1546.2 (13) Å^3^
*Z* = 4
Mo *K*α radiation
μ = 2.67 mm^−1^
*T* = 298 K
0.49 × 0.34 × 0.24 mm

#### Data collection

Bruker APEX diffractometer
Absorption correction: multi-scan (*SADABS*; Bruker, 2002 ▶) *T*_min_ = 0.355, *T*_max_ = 0.567
7968 measured reflections
5326 independent reflections
3031 reflections with *I* > 2σ(*I*)
*R*_int_ = 0.020

#### Refinement

*R*[*F* ^2^ > 2σ(*F* ^2^)] = 0.049
*wR*(*F* ^2^) = 0.135
*S* = 1.00
5326 reflections
383 parameters
H-atom parameters constrained
Δρ_max_ = 0.60 e Å^−3^
Δρ_min_ = −0.75 e Å^−3^

### 

Data collection: *SMART* (Bruker, 2002 ▶); cell refinement: *SAINT* (Bruker, 2002 ▶); data reduction: *SAINT*; program(s) used to solve structure: *SHELXS97* (Sheldrick, 2008 ▶); program(s) used to refine structure: *SHELXL97* (Sheldrick, 2008 ▶); molecular graphics: *SHELXTL* (Sheldrick, 2008 ▶); software used to prepare material for publication: *SHELXL97*.

## Supplementary Material

Crystal structure: contains datablock(s) I, global. DOI: 10.1107/S1600536812006046/zq2148sup1.cif

Structure factors: contains datablock(s) I. DOI: 10.1107/S1600536812006046/zq2148Isup2.hkl

Supplementary material file. DOI: 10.1107/S1600536812006046/zq2148Isup3.cml

Additional supplementary materials:  crystallographic information; 3D view; checkCIF report

## Figures and Tables

**Table 1:** Hydrogen-bond geometry (Å, °)

*D*—H⋯*A*	*D*—H	H⋯*A*	*D*⋯*A*	*D*—H⋯*A*
C29—H29⋯O2^i^	0.93	2.59	3.256 (5)	129
C33—H33*A*⋯O1^ii^	0.96	2.46	3.323 (5)	149

Symmetry codes: (i) 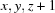; (ii) 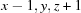.

**Fig. 1.**
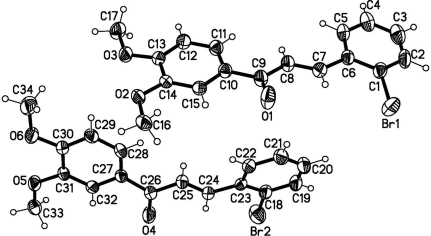
The molecular structure of the title compound with displacement ellipsoids drawn at the 30% probability level. H atoms are shown as spheres of arbitrary size.

**Fig. 2.**
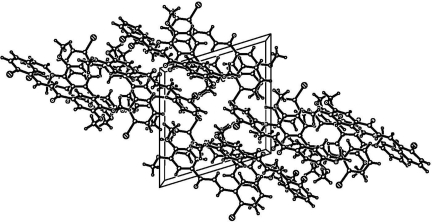
Packing diagram of the title compound viewed along the crystallographic b axis.

## supplementary crystallographic information

### Comment

Natural chalcones exist widely in vegetables, fruits, medicinal plants, and so on. Natural and synthetical chalcones have extensive biological properties such as anti-inflammatory, antitumor, antioxidant (Nielsen *et al.*, 2005; Wu *et al.*, 2010, 2011*a*,*b*). We synthesized a series of chalcones in order to study antitumor activity. We obtain monocrystals of the title compound, and its crystal structure was characterized by a X-ray diffraction study.

The crystal structure of the title compound, C_17_H_15_BrO_3_, a chalcone derivative, exhibits two crystallographically independent molecules per asymmetric unit showing an *E* configuration about the ethylene double bond (see for instance: Wu *et al.*, 2009; Peng *et al.*, 2010; Huang *et al.*, 2010). In each molecule, the two phenyl rings are almost coplanar: the mean planes make dihedral angles of 9.3 (2) and 19.4 (2)°. In the crystal, molecules are linked through weak intermolecular C—H···O hydrogen bonds.

### Experimental

The title compound was synthesized by Claisene–Schmidt condensation between 3,4-dimethoxybenzaldehyde and 2'-bromoacetophenone. 3,4-Dimethoxybenzaldehyde (1 mmol) and 2'-bromoacetophenone (1 mmol) were dissolved in ethanol (10 ml). NaOH (40%, 5 drops) was added at 283 K. The temperature during the whole reaction was controlled to remain below 288 K. The reaction was monitored by thin-layer chromatography. After reaction was over, 10 ml H_2_O was added and the white solid was precipitated, washed with a mixture of water and cold ethanol (10:1), and dried (yield: 75.3%, m.p. 372–376 K). The title compound was dissolved in a mixture solution of ethanol and dichloromethane. Single crystals were obtained by solvent evaporation.

### Refinement

All hydrogen positions were calculated after each cycle of refinement using a riding model, with C—H = 0.93 Å and *U*_iso_(H) = 1.2*U*_eq_(C) for aromatic H atoms, and with C—H = 0.96 Å and *U*_iso_(H) = 1.5*U*_eq_(C) for methyl H atoms.

### Crystal data

**Table:**

C_17_H_15_BrO_3_	*Z* = 4
*M**_r_* = 347.20	*F*(000) = 704
Triclinic, *P*1	*D*_x_ = 1.492 Mg m^−^^3^
Hall symbol: -P 1	Mo *K*α radiation, λ = 0.71073 Å
*a* = 11.574 (6) Å	Cell parameters from 2084 reflections
*b* = 11.781 (6) Å	θ = 2.4–22.1°
*c* = 11.877 (6) Å	µ = 2.67 mm^−^^1^
α = 91.857 (9)°	*T* = 298 K
β = 107.021 (9)°	Block, colourless
γ = 91.917 (9)°	0.49 × 0.34 × 0.24 mm
*V* = 1546.2 (13) Å^3^	

### Data collection

**Table:**

Bruker APEX diffractometer	5326 independent reflections
Radiation source: fine-focus sealed tube	3031 reflections with *I* > 2σ(*I*)
Graphite monochromator	*R*_int_ = 0.020
φ and ω scans	θ_max_ = 25.0°, θ_min_ = 1.7°
Absorption correction: multi-scan (*SADABS*; Bruker, 2002)	*h* = −13→7
*T*_min_ = 0.355, *T*_max_ = 0.567	*k* = −13→14
7968 measured reflections	*l* = −13→14

### Refinement

**Table:**

Refinement on *F*^2^	Primary atom site location: structure-invariant direct methods
Least-squares matrix: full	Secondary atom site location: difference Fourier map
*R*[*F*^2^ > 2σ(*F*^2^)] = 0.049	Hydrogen site location: inferred from neighbouring sites
*wR*(*F*^2^) = 0.135	H-atom parameters constrained
*S* = 1.00	*w* = 1/[σ^2^(*F*_o_^2^) + (0.0693*P*)^2^] where *P* = (*F*_o_^2^ + 2*F*_c_^2^)/3
5326 reflections	(Δ/σ)_max_ = 0.001
383 parameters	Δρ_max_ = 0.60 e Å^−^^3^
0 restraints	Δρ_min_ = −0.75 e Å^−^^3^

### Special details

**Table:**

Geometry. All e.s.d.'s (except the e.s.d. in the dihedral angle between two l.s. planes) are estimated using the full covariance matrix. The cell e.s.d.'s are taken into account individually in the estimation of e.s.d.'s in distances, angles and torsion angles; correlations between e.s.d.'s in cell parameters are only used when they are defined by crystal symmetry. An approximate (isotropic) treatment of cell e.s.d.'s is used for estimating e.s.d.'s involving l.s. planes.
Refinement. Refinement of *F*^2^ against ALL reflections. The weighted *R*-factor *wR* and goodness of fit *S* are based on *F*^2^, conventional *R*-factors *R* are based on *F*, with *F* set to zero for negative *F*^2^. The threshold expression of *F*^2^ > σ(*F*^2^) is used only for calculating *R*-factors(gt) *etc*. and is not relevant to the choice of reflections for refinement. *R*-factors based on *F*^2^ are statistically about twice as large as those based on *F*, and *R*-factors based on ALL data will be even larger.

### Fractional atomic coordinates and isotropic or equivalent isotropic displacement parameters (Å^2^)

**Table:**

	*x*	*y*	*z*	*U*_iso_*/*U*_eq_	
Br1	1.31399 (5)	0.24675 (5)	0.31891 (5)	0.1117 (3)	
Br2	0.65864 (5)	0.47379 (5)	1.48067 (4)	0.1092 (3)	
O1	0.9383 (3)	0.2110 (3)	−0.0037 (3)	0.0994 (11)	
O2	0.5144 (2)	0.1793 (2)	−0.2772 (2)	0.0612 (7)	
O3	0.4199 (2)	−0.0087 (2)	−0.2365 (2)	0.0663 (7)	
O4	0.3197 (2)	0.4779 (3)	1.1131 (3)	0.0945 (10)	
O5	−0.0283 (2)	0.3636 (2)	0.7519 (2)	0.0674 (7)	
O6	0.0593 (2)	0.2352 (2)	0.6217 (2)	0.0712 (8)	
C1	1.2701 (3)	0.0977 (4)	0.3508 (3)	0.0687 (11)	
C2	1.3450 (4)	0.0455 (5)	0.4462 (4)	0.0872 (15)	
H2	1.4151	0.0837	0.4931	0.105*	
C3	1.3160 (4)	−0.0620 (6)	0.4713 (4)	0.0916 (15)	
H3	1.3671	−0.0972	0.5348	0.110*	
C4	1.2123 (4)	−0.1185 (4)	0.4041 (4)	0.0833 (13)	
H4	1.1925	−0.1916	0.4217	0.100*	
C5	1.1384 (3)	−0.0663 (4)	0.3111 (3)	0.0669 (11)	
H5	1.0687	−0.1058	0.2652	0.080*	
C6	1.1626 (3)	0.0426 (3)	0.2819 (3)	0.0564 (10)	
C7	1.0796 (3)	0.0964 (3)	0.1822 (3)	0.0611 (10)	
H7	1.1088	0.1627	0.1576	0.073*	
C8	0.9686 (3)	0.0613 (3)	0.1239 (3)	0.0618 (10)	
H8	0.9355	−0.0046	0.1454	0.074*	
C9	0.8956 (3)	0.1246 (4)	0.0248 (3)	0.0625 (10)	
C10	0.7700 (3)	0.0834 (3)	−0.0386 (3)	0.0535 (9)	
C11	0.7197 (3)	−0.0189 (3)	−0.0212 (3)	0.0614 (10)	
H11	0.7648	−0.0658	0.0351	0.074*	
C12	0.6032 (3)	−0.0537 (3)	−0.0856 (3)	0.0605 (10)	
H12	0.5702	−0.1234	−0.0727	0.073*	
C13	0.5364 (3)	0.0158 (3)	−0.1691 (3)	0.0515 (9)	
C14	0.5866 (3)	0.1193 (3)	−0.1896 (3)	0.0473 (8)	
C15	0.7013 (3)	0.1528 (3)	−0.1241 (3)	0.0524 (9)	
H15	0.7342	0.2227	−0.1366	0.063*	
C16	0.5611 (4)	0.2853 (3)	−0.3024 (4)	0.0765 (12)	
H16A	0.5780	0.3361	−0.2342	0.115*	
H16B	0.5025	0.3173	−0.3670	0.115*	
H16C	0.6342	0.2740	−0.3232	0.115*	
C17	0.3661 (4)	−0.1170 (4)	−0.2221 (4)	0.0778 (13)	
H17A	0.4091	−0.1768	−0.2464	0.117*	
H17B	0.2830	−0.1222	−0.2696	0.117*	
H17C	0.3701	−0.1243	−0.1408	0.117*	
C18	0.7467 (3)	0.4225 (3)	1.3779 (3)	0.0659 (11)	
C19	0.8659 (4)	0.4014 (4)	1.4280 (4)	0.0818 (13)	
H19	0.9010	0.4123	1.5089	0.098*	
C20	0.9328 (4)	0.3639 (4)	1.3572 (5)	0.0896 (15)	
H20	1.0139	0.3494	1.3907	0.108*	
C21	0.8821 (4)	0.3475 (4)	1.2382 (5)	0.0785 (12)	
H21	0.9288	0.3234	1.1910	0.094*	
C22	0.7612 (3)	0.3668 (3)	1.1886 (4)	0.0628 (10)	
H22	0.7268	0.3537	1.1079	0.075*	
C23	0.6898 (3)	0.4055 (3)	1.2563 (3)	0.0555 (9)	
C24	0.5611 (3)	0.4248 (3)	1.2031 (3)	0.0579 (9)	
H24	0.5237	0.4667	1.2490	0.069*	
C25	0.4934 (3)	0.3899 (3)	1.0985 (3)	0.0577 (10)	
H25	0.5277	0.3484	1.0496	0.069*	
C26	0.3628 (3)	0.4145 (3)	1.0551 (3)	0.0577 (10)	
C27	0.2870 (3)	0.3600 (3)	0.9424 (3)	0.0511 (9)	
C28	0.3319 (3)	0.2920 (3)	0.8702 (3)	0.0555 (9)	
H28	0.4133	0.2757	0.8939	0.067*	
C29	0.2586 (3)	0.2475 (3)	0.7631 (3)	0.0604 (10)	
H29	0.2905	0.2007	0.7161	0.072*	
C30	0.1385 (3)	0.2725 (3)	0.7262 (3)	0.0525 (9)	
C31	0.0910 (3)	0.3418 (3)	0.7983 (3)	0.0524 (9)	
C32	0.1640 (3)	0.3843 (3)	0.9051 (3)	0.0533 (9)	
H32	0.1319	0.4294	0.9532	0.064*	
C33	−0.0848 (3)	0.4297 (3)	0.8225 (3)	0.0666 (11)	
H33A	−0.0833	0.3903	0.8923	0.100*	
H33B	−0.1671	0.4411	0.7783	0.100*	
H33C	−0.0419	0.5020	0.8442	0.100*	
C34	0.1004 (4)	0.1567 (5)	0.5498 (4)	0.1024 (17)	
H34A	0.1655	0.1919	0.5268	0.154*	
H34B	0.0351	0.1346	0.4808	0.154*	
H34C	0.1283	0.0907	0.5934	0.154*	

### Atomic displacement parameters (Å^2^)

**Table:**

	*U*^11^	*U*^22^	*U*^33^	*U*^12^	*U*^13^	*U*^23^
Br1	0.0906 (4)	0.0923 (4)	0.1373 (5)	−0.0230 (3)	0.0170 (3)	−0.0320 (3)
Br2	0.1124 (4)	0.1390 (6)	0.0687 (3)	0.0344 (4)	0.0123 (3)	−0.0010 (3)
O1	0.0711 (19)	0.102 (3)	0.102 (2)	−0.0269 (18)	−0.0118 (16)	0.0457 (19)
O2	0.0512 (14)	0.0496 (16)	0.0749 (17)	0.0054 (12)	0.0047 (12)	0.0129 (13)
O3	0.0526 (15)	0.0552 (17)	0.0806 (18)	−0.0091 (13)	0.0041 (13)	0.0067 (13)
O4	0.0570 (17)	0.116 (3)	0.093 (2)	0.0218 (17)	−0.0012 (15)	−0.0437 (19)
O5	0.0446 (14)	0.083 (2)	0.0687 (16)	0.0124 (13)	0.0087 (12)	−0.0123 (14)
O6	0.0571 (15)	0.090 (2)	0.0599 (16)	0.0101 (14)	0.0076 (13)	−0.0165 (14)
C1	0.054 (2)	0.087 (3)	0.060 (2)	0.002 (2)	0.012 (2)	−0.023 (2)
C2	0.047 (3)	0.130 (5)	0.069 (3)	0.010 (3)	−0.003 (2)	−0.027 (3)
C3	0.069 (3)	0.138 (5)	0.064 (3)	0.024 (3)	0.012 (2)	0.012 (3)
C4	0.062 (3)	0.113 (4)	0.074 (3)	0.014 (3)	0.016 (2)	0.026 (3)
C5	0.050 (2)	0.088 (3)	0.056 (2)	0.005 (2)	0.0049 (19)	0.010 (2)
C6	0.047 (2)	0.071 (3)	0.049 (2)	0.0059 (19)	0.0117 (17)	−0.0051 (19)
C7	0.059 (2)	0.063 (3)	0.059 (2)	0.0018 (19)	0.0133 (19)	−0.0015 (19)
C8	0.053 (2)	0.069 (3)	0.058 (2)	0.000 (2)	0.0078 (19)	0.0084 (19)
C9	0.056 (2)	0.069 (3)	0.057 (2)	0.002 (2)	0.0077 (19)	0.010 (2)
C10	0.052 (2)	0.056 (2)	0.051 (2)	0.0005 (18)	0.0124 (17)	0.0058 (18)
C11	0.063 (2)	0.064 (3)	0.053 (2)	0.004 (2)	0.0094 (19)	0.0086 (19)
C12	0.062 (2)	0.056 (2)	0.060 (2)	−0.003 (2)	0.013 (2)	0.0102 (19)
C13	0.048 (2)	0.052 (2)	0.053 (2)	0.0002 (18)	0.0128 (17)	0.0003 (17)
C14	0.047 (2)	0.044 (2)	0.050 (2)	0.0053 (17)	0.0110 (16)	0.0036 (16)
C15	0.053 (2)	0.049 (2)	0.055 (2)	0.0006 (17)	0.0136 (18)	0.0067 (17)
C16	0.074 (3)	0.048 (3)	0.095 (3)	0.007 (2)	0.002 (2)	0.021 (2)
C17	0.068 (3)	0.069 (3)	0.091 (3)	−0.021 (2)	0.017 (2)	0.005 (2)
C18	0.061 (2)	0.059 (3)	0.067 (3)	−0.003 (2)	0.002 (2)	0.007 (2)
C19	0.068 (3)	0.075 (3)	0.080 (3)	0.000 (2)	−0.014 (3)	0.007 (2)
C20	0.057 (3)	0.074 (3)	0.119 (4)	0.002 (2)	−0.004 (3)	0.014 (3)
C21	0.053 (3)	0.067 (3)	0.112 (4)	−0.002 (2)	0.021 (3)	0.005 (3)
C22	0.052 (2)	0.055 (3)	0.076 (3)	−0.0033 (19)	0.011 (2)	0.001 (2)
C23	0.049 (2)	0.040 (2)	0.068 (2)	−0.0061 (17)	0.0037 (19)	0.0036 (18)
C24	0.050 (2)	0.054 (2)	0.063 (2)	0.0038 (18)	0.0073 (18)	−0.0033 (18)
C25	0.046 (2)	0.057 (2)	0.064 (2)	0.0010 (17)	0.0074 (18)	0.0017 (19)
C26	0.048 (2)	0.059 (3)	0.063 (2)	0.0063 (19)	0.0101 (19)	0.0017 (19)
C27	0.044 (2)	0.049 (2)	0.057 (2)	0.0021 (17)	0.0105 (17)	0.0057 (17)
C28	0.0420 (19)	0.064 (3)	0.061 (2)	0.0125 (18)	0.0138 (18)	0.0076 (19)
C29	0.054 (2)	0.067 (3)	0.061 (2)	0.0157 (19)	0.0173 (19)	−0.0037 (19)
C30	0.048 (2)	0.055 (2)	0.052 (2)	0.0037 (18)	0.0106 (17)	0.0018 (17)
C31	0.0361 (19)	0.058 (2)	0.061 (2)	0.0074 (17)	0.0096 (17)	0.0031 (18)
C32	0.047 (2)	0.051 (2)	0.061 (2)	0.0058 (17)	0.0146 (18)	−0.0018 (18)
C33	0.042 (2)	0.077 (3)	0.081 (3)	0.0096 (19)	0.0188 (19)	0.002 (2)
C34	0.096 (3)	0.126 (5)	0.075 (3)	0.026 (3)	0.012 (3)	−0.032 (3)

### Geometric parameters (Å, º)

**Table:**

Br1—C1	1.892 (5)	C16—H16A	0.9600
Br2—C18	1.902 (4)	C16—H16B	0.9600
O1—C9	1.217 (4)	C16—H16C	0.9600
O2—C14	1.363 (4)	C17—H17A	0.9600
O2—C16	1.419 (4)	C17—H17B	0.9600
O3—C13	1.367 (4)	C17—H17C	0.9600
O3—C17	1.438 (4)	C18—C19	1.367 (6)
O4—C26	1.214 (4)	C18—C23	1.405 (5)
O5—C31	1.365 (4)	C19—C20	1.372 (7)
O5—C33	1.432 (4)	C19—H19	0.9300
O6—C30	1.358 (4)	C20—C21	1.367 (6)
O6—C34	1.423 (5)	C20—H20	0.9300
C1—C2	1.386 (6)	C21—C22	1.379 (5)
C1—C6	1.399 (5)	C21—H21	0.9300
C2—C3	1.364 (6)	C22—C23	1.388 (5)
C2—H2	0.9300	C22—H22	0.9300
C3—C4	1.368 (6)	C23—C24	1.466 (5)
C3—H3	0.9300	C24—C25	1.304 (4)
C4—C5	1.362 (5)	C24—H24	0.9300
C4—H4	0.9300	C25—C26	1.489 (5)
C5—C6	1.380 (5)	C25—H25	0.9300
C5—H5	0.9300	C26—C27	1.481 (5)
C6—C7	1.466 (5)	C27—C28	1.375 (5)
C7—C8	1.315 (5)	C27—C32	1.404 (5)
C7—H7	0.9300	C28—C29	1.381 (5)
C8—C9	1.474 (5)	C28—H28	0.9300
C8—H8	0.9300	C29—C30	1.375 (5)
C9—C10	1.484 (5)	C29—H29	0.9300
C10—C11	1.368 (5)	C30—C31	1.401 (5)
C10—C15	1.399 (5)	C31—C32	1.369 (4)
C11—C12	1.383 (5)	C32—H32	0.9300
C11—H11	0.9300	C33—H33A	0.9600
C12—C13	1.378 (5)	C33—H33B	0.9600
C12—H12	0.9300	C33—H33C	0.9600
C13—C14	1.392 (5)	C34—H34A	0.9600
C14—C15	1.365 (4)	C34—H34B	0.9600
C15—H15	0.9300	C34—H34C	0.9600
			
C14—O2—C16	117.9 (3)	H17A—C17—H17C	109.5
C13—O3—C17	117.2 (3)	H17B—C17—H17C	109.5
C31—O5—C33	118.1 (3)	C19—C18—C23	122.2 (4)
C30—O6—C34	117.9 (3)	C19—C18—Br2	117.1 (3)
C2—C1—C6	120.6 (4)	C23—C18—Br2	120.7 (3)
C2—C1—Br1	118.6 (3)	C18—C19—C20	119.0 (4)
C6—C1—Br1	120.8 (3)	C18—C19—H19	120.5
C3—C2—C1	119.9 (4)	C20—C19—H19	120.5
C3—C2—H2	120.0	C21—C20—C19	121.0 (4)
C1—C2—H2	120.0	C21—C20—H20	119.5
C2—C3—C4	120.6 (4)	C19—C20—H20	119.5
C2—C3—H3	119.7	C20—C21—C22	119.6 (4)
C4—C3—H3	119.7	C20—C21—H21	120.2
C5—C4—C3	119.2 (5)	C22—C21—H21	120.2
C5—C4—H4	120.4	C21—C22—C23	121.6 (4)
C3—C4—H4	120.4	C21—C22—H22	119.2
C4—C5—C6	122.8 (4)	C23—C22—H22	119.2
C4—C5—H5	118.6	C22—C23—C18	116.5 (3)
C6—C5—H5	118.6	C22—C23—C24	121.3 (3)
C5—C6—C1	116.8 (3)	C18—C23—C24	122.2 (4)
C5—C6—C7	121.2 (3)	C25—C24—C23	127.3 (4)
C1—C6—C7	122.0 (4)	C25—C24—H24	116.3
C8—C7—C6	127.2 (4)	C23—C24—H24	116.3
C8—C7—H7	116.4	C24—C25—C26	122.1 (4)
C6—C7—H7	116.4	C24—C25—H25	119.0
C7—C8—C9	121.1 (4)	C26—C25—H25	119.0
C7—C8—H8	119.5	O4—C26—C27	121.0 (3)
C9—C8—H8	119.5	O4—C26—C25	119.6 (3)
O1—C9—C8	119.7 (3)	C27—C26—C25	119.4 (3)
O1—C9—C10	120.5 (3)	C28—C27—C32	118.7 (3)
C8—C9—C10	119.8 (3)	C28—C27—C26	123.6 (3)
C11—C10—C15	118.7 (3)	C32—C27—C26	117.7 (3)
C11—C10—C9	124.2 (3)	C27—C28—C29	121.4 (3)
C15—C10—C9	117.1 (3)	C27—C28—H28	119.3
C10—C11—C12	121.3 (4)	C29—C28—H28	119.3
C10—C11—H11	119.3	C30—C29—C28	119.8 (3)
C12—C11—H11	119.3	C30—C29—H29	120.1
C13—C12—C11	119.4 (4)	C28—C29—H29	120.1
C13—C12—H12	120.3	O6—C30—C29	124.6 (3)
C11—C12—H12	120.3	O6—C30—C31	115.8 (3)
O3—C13—C12	124.6 (3)	C29—C30—C31	119.7 (3)
O3—C13—C14	115.2 (3)	O5—C31—C32	125.1 (3)
C12—C13—C14	120.2 (3)	O5—C31—C30	114.8 (3)
O2—C14—C15	125.1 (3)	C32—C31—C30	120.1 (3)
O2—C14—C13	115.4 (3)	C31—C32—C27	120.3 (3)
C15—C14—C13	119.5 (3)	C31—C32—H32	119.8
C14—C15—C10	120.9 (3)	C27—C32—H32	119.8
C14—C15—H15	119.6	O5—C33—H33A	109.5
C10—C15—H15	119.6	O5—C33—H33B	109.5
O2—C16—H16A	109.5	H33A—C33—H33B	109.5
O2—C16—H16B	109.5	O5—C33—H33C	109.5
H16A—C16—H16B	109.5	H33A—C33—H33C	109.5
O2—C16—H16C	109.5	H33B—C33—H33C	109.5
H16A—C16—H16C	109.5	O6—C34—H34A	109.5
H16B—C16—H16C	109.5	O6—C34—H34B	109.5
O3—C17—H17A	109.5	H34A—C34—H34B	109.5
O3—C17—H17B	109.5	O6—C34—H34C	109.5
H17A—C17—H17B	109.5	H34A—C34—H34C	109.5
O3—C17—H17C	109.5	H34B—C34—H34C	109.5

### Hydrogen-bond geometry (Å, º)

**Table:**

*D*—H···*A*	*D*—H	H···*A*	*D*···*A*	*D*—H···*A*
C29—H29···O2^i^	0.93	2.59	3.256 (5)	129
C33—H33*A*···O1^ii^	0.96	2.46	3.323 (5)	149

Symmetry codes: (i) *x*, *y*, *z*+1; (ii) *x*−1, *y*, *z*+1.
